# Assessing transferability in systematic reviews of health economic evaluations – a review of methodological guidance

**DOI:** 10.1186/s12874-022-01536-6

**Published:** 2022-02-20

**Authors:** Alina Weise, Roland Brian Büchter, Dawid Pieper, Tim Mathes

**Affiliations:** 1 Institute for Research in Operative Medicine, Faculty of Health—School of Medicine, Witten/Herdecke University, Cologne, Germany; 2 Faculty of Health Sciences Brandenburg, Brandenburg Medical School Theodor Fontane, Institute for Health Services and Health System Research, Neuruppin, Germany; 3 Center for Health Services Research, Brandenburg Medical School Theodor Fontane, Rüdersdorf, Germany; 4 Department for Medical Statistics, University Medical Centre Goettingen, Goettingen, Germany

**Keywords:** Transferability, External validity, Generalisability, Applicability, Health economic evaluations, Methods, Health technology assessment

## Abstract

**Objective:**

For assessing cost-effectiveness, Health Technology Assessment (HTA) organisations may use primary economic evaluations (P-HEs) or Systematic Reviews of Health Economic evaluations (SR-HEs). A prerequisite for meaningful results of SR-HEs is that the results from existing P-HEs are transferable to the decision context (e.g, HTA jurisdiction). A particularly pertinent issue is the high variability of costs and resource needs across jurisdictions. Our objective was to review the methods documents of HTA organisations and compare their recommendations on considering transferability in SR-HE.

**Methods:**

We systematically hand searched the webpages of 158 HTA organisations for relevant methods documents from 8th January to 31st March 2019. Two independent reviewers performed searches and selected documents according to pre-defined criteria. One reviewer extracted data in standardised and piloted tables and a second reviewer checked them for accuracy. We synthesised data using tabulations and in a narrative way.

**Results:**

We identified 155 potentially relevant documents from 63 HTA organisations. Of these, 7 were included in the synthesis. The included organisations have different aims when preparing a SR-HE (e.g. to determine the need for conducting their own P-HE). The recommendations vary regarding the underlying terminology (e.g. transferability/generalisability), the assessment approaches (e.g. structure), the assessment criteria and the integration in the review process.

**Conclusion:**

Only few HTA organisations address the assessment of transferability in their methodological recommendations for SR-HEs. Transferability considerations are related to different purposes. The assessment concepts and criteria are heterogeneous. Developing standards to consider transferability in SR-HEs is desirable.

**Supplementary Information:**

The online version contains supplementary material available at 10.1186/s12874-022-01536-6.

## Introduction

Economic evaluations play an important role when making pricing and reimbursement decisions on health technologies. To support these decisions Health Technology Assessments (HTAs) often include cost-effectiveness data [1]. These can be based on primary economic evaluations (P-HEs) or Systematic Reviews of Health Economic evaluations (SR-HEs) [2]. P-HEs are time and resource intensive. As not all decision-makers have the ability to commission de-novo analyses, decisions may need to rely on existing similar studies carried out somewhere else. SR-HEs have the potential to meet the increasing demand on incorporating cost-effectiveness considerations [3]. Another possible advantage is the ability to assess consistency between P-HEs, and that a higher confidence may be placed in the results from SR-HEs compared to a single P-HE. That said, a prerequisite to meaningful SR-HEs is that the results from included P-HEs are transferable to the context of interest (e.g. between jurisdictions or over time). But - especially due to concerns regarding the transferability of P-HEs - the usefulness of SR-HEs has been questioned [2, 3]. A P-HE would be considered transferable, if a similar level of cost-effectiveness of the invention could be achieved in the context of interest [4]. However, in the worst case no P-HE can be identified that would be transferable to the own context. In addition, a P-HE specifically developed for the own context will always be better applicable than previously developed P-HEs (suited for different jurisdictions), assuming that data and methodological quality are consistent.

There are many reasons why health economic evaluations might not be transferable between different decision contexts, e.g. regarding methodological aspects [5], geographical settings or time periods [2, 6, 7]. Therefore, considering transferability of the results from included P-HEs is important when conducting a SR-HE [8]. Several tools for assessing transferability of P-HEs have been developed and suggested [6, 9–11], but there is no widely agreed approach [12, 13].

Our objective was to review the methods documents of HTA organisations in regard to determine how transferability is recommended to be considered when performing SR-HEs in the context of HTAs. We specifically focussed on methods for assessing transferability in SR-HEs, as methods and positions on the transferability of decision model input data are already addressed elsewhere [6, 14]. Moreover, we focused our analysis on HTA organisations because they prepare recommendations for specific jurisdictions, which makes transferability issues particularly relevant.

This is the second part of a larger research project of our team on this topic. In the first part we reviewed the methodological recommendations of evidence synthesis producing organisations on assessing context suitability (e.g. transferability) of evidence on effectiveness [15].

## Methods

This review is reported according to the Preferred Reporting Items for Systematic Reviews and Meta-Analysis (PRISMA 2020 Statement) [16], in so far as it is applicable to methodological research.

There was no published protocol for this review. Unless otherwise indicated, we specified all described methods in advance.

### Search strategy

We performed structured searches on the webpages of HTA organisations. In a first step, we identified HTA organisations through publicly available member lists of the following HTA umbrella organisations: Health Technology Assessment international (HTAi), International Agency of Health Technology Assessment (INAHTA), European Network of HTA Agencies (EUnetHTA) and Red de Evaluación de Tecnologías en Salud de las Américas (RedETSA). In a second step, two independent reviewers performed structured searches on the webpages of identified HTA organisations. The searches were performed from 8th January to 31st March 2019. To allow for a thorough search on webpages with different and sometimes complex site-structures, we checked each section of the webpages carefully. We used machine based and browser translation tools to identify English or German language documents on foreign language websites. Both reviewers downloaded and stored identified documents, independently. The list of identified documents was compared and synchronised manually. In cases where several versions of one document existed, only the latest version was considered. We removed duplicates manually.

### Eligibility criteria and screening

We screened all identified documents against the following pre-defined eligibility criteria:

(i) Publication type: Methods documents for the preparation of HTAs (e.g. guidelines, handbooks, manuals, standard operation procedures)
(ii) Documents include recommendations for appraising transferability when conducting SR-HEs
(iii) The appraisal process is specified, e.g. in form of concrete methods, questionnaires or tools
(iv) Recommendations on external validity, generalisability, extrapolation, transferability or applicability are considered (according to the definitions of Burford et al. [4])
(v) Languages: English, German

There is no consensus on the terminology of “transferability” and other related terms such as “generalisability” are sometimes used interchangeably [4, 10]. According to Burford et al. we define transferability as to “whether when implementing an intervention in a particular setting or population, the level of [cost]-effectiveness of the intervention (i.e., the effect size) will be similar to that observed in the systematic review.” [4] However, due to heterogeneity we considered all related terms, which are defined in Table 1. Moreover, we decided to reassign the different terms and definitions to the corresponding terminology of Burford et al. [4]. This was an important step when synthesising evidence, to archive a uniform terminology and to distinguish heterogeneity related to terminology from heterogeneity related to other aspects.

**Table 1:** Terms and definitions^a^

Term	Definition/explanation
Types of considered health technologies	Describes whether the methods document relates to a specific type of intervention (pharmaceuticals, medical devices, medical services (procedures, diagnostics, public health interventions) or whether it is generic
Review Purpose	Describes the objective pursued by the preparation of the SR-HE
Original terminology and definition	Describes which transferability related term was used in the methods document (eg, external validity, generalisability, applicability, transferability) and how it is defined by the organisation
Harmonised terminology and definition	Due to the high heterogeneity in the terminology we decided to reassign the different definitions in the methods documents to the corresponding definition by Burford et al. (2013) to achieve a uniform terminology
*Applicability according to Burford et al.* [4]	“Whether the findings of a review can be applied in a particular context or population. This includes the consideration of the feasibility of implementing the intervention and variation in intervention fidelity, population characteristics, context, culture, values, and preferences”
*External validity/ generalizability according to Burford et al.* [4]	“The extent to which results provide a correct basis for generalizations to other circumstances”
*Transferability according to Burford et al* [4]	“Whether when implementing an intervention in a particular setting or population, the level of effectiveness of the intervention (ie, the effect size) will be similar to that observed in the systematic review”
Assessment approach concepts	Describes how the transferability assessment is operationalised. This includes the integration of assessment in the review preparation process, the target data addressed by the transferability assessment, the structuring of the assessment approach, the provision of guidance on completion, the integration of assessment in the quality of evidence rating and the use of results derived from sensitivity analyses
*Integration of assessment in the preparation process*	Transferability considerations can be addressed at different steps of the systematic review preparation process. It can be considered in study selection, in study assessment, or both
In study selection	Consideration of transferability in study selection, when defining and applying eligibility criteria
In study assessment	Assessment of transferability of included evidence
*Target data*	Describes for which data (effectiveness data, cost data, or both) transferability should be assessed
*Structuring of the assessment approach*	Describes, whether the transferability consideration follows a clear structure or not. We differentiate between structured and non-structured approaches
Non-structured	The transferability assessment does not follow a clear structure
Structured	The transferability assessment follows a structure and may comprise different steps, a checklist or questions that have to be followed or rated
*Guidance on completion*	Describes, whether the methods document provides instructions and/or item descriptions (eg, definitions, examples) which can be used by assessors as a guide, when assessing transferability
*Combination of different assessments*	Describes, whether the assessment of transferability is combined with other aspects for assessing quality of evidence or not. We differentiate between standalone and combined assessments
Standalone assessment	Transferability is assessed independently from other aspects for assessing quality of evidence
Combined assessment	The assessment of transferability is combined with other aspects for assessing quality of evidence (methodological study quality)
Assessment criteria	Describes which factors might affect transferability and are recommended to be considered, when assessing transferability. These criteria relate to the following domains: PICO (population, intervention, comparison, outcome), health system, clinical practice, costs, methodological aspects, other

^a^Adapted from Weise et al. 2020 [15]

Two independent reviewers performed full-text screening. Discrepancies were resolved by discussion and arbitration as necessary.

### Data extraction

We performed data extraction using standardised and piloted data extraction sheets. We developed the data extraction sheets inductively, by reviewing included methods documents. We piloted the extraction sheets on a sample of included documents. Final data extraction was performed by one reviewer and checked by a second reviewer. We resolved discrepancies by discussion, and arbitration if necessary. Data were extracted verbatim to avoid interpretation bias.

We collected information on the following issues: the types of considered health technologies, the terminology, the purpose of the SR-HE, the recommended assessment approach and the assessment criteria. All data extraction items are defined in Table 1.

Evidence was synthesised using tabulations and in a narrative way.

## Results

### Literature search

**Fig. 1:**
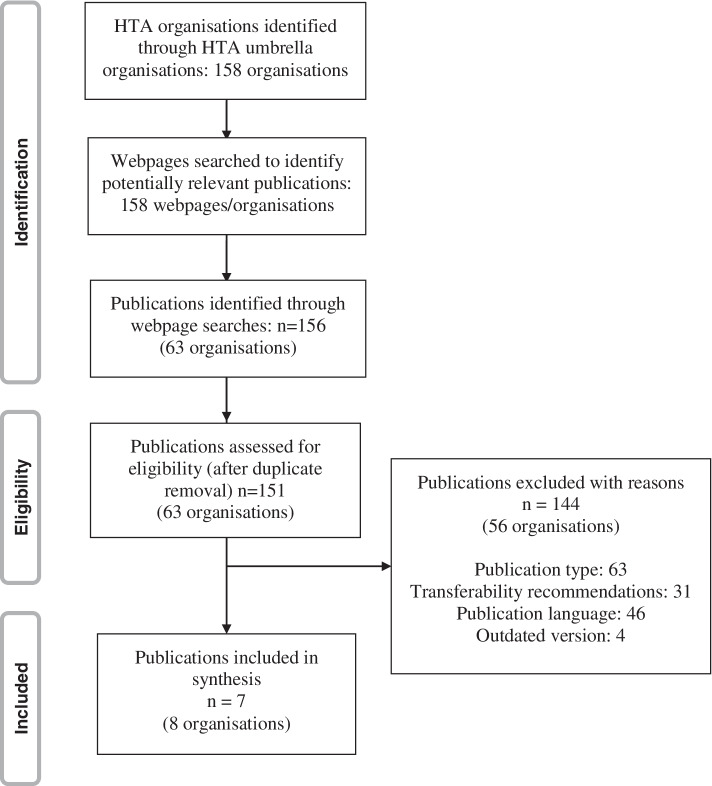
PRISMA Flow-Chart of document selection

We identified 158 HTA organisations. Webpage searches resulted in 156 potentially relevant publications from 63 of these organisations. After deduplication, a total of 151 publications remained and were assessed for eligibility. Seven documents from eight organisations were included in our synthesis. Two organisations (Gesundheit Österreich GmbH (GÖG)/Ludwig Boltzmann Institut (LBI)) share the same methods document and are therefore considered together in the following. The selection process is illustrated in Fig. 1. We provide a list of excluded documents as supplement (see Additional file 1).

We included methods documents from the following organisations: Agency for Care Effectiveness (ACE) [17], European Network for Health Technology Assessment (EUnetHTA) [18], GÖG/ LBI [19], Health Information and Quality Authority (HIQA) [20], Health Quality Ontario (HQA) [21], National Institute for Health and Care Excellence (NICE) [22], Swedish Agency for Health Technology Assessment and Assessment of Social Services (SBU) [23].

### Guidance characteristics

Table 2 provides an overview of the characteristics of included methods documents, including the types of considered health technologies (e.g. non-pharmaceuticals) and the transferability-related terminology. The organisations mainly use the terms transferability, applicability and generalisability with varying and sometimes missing definitions.

**Table 2:** Guidance characteristics

Organisation, Country	Title, year	Considered health technologies	Terminology	Definition	Harmonised Terminology according to Burford et al. [4]
ACE Agency for care effectiveness, Singapore [17]	Medical Technologies Evaluation Methods and Process Guide Version 1.0, 2018	non-pharmaceuticals (e.g. medical devices, diagnostics, medical services/ procedures)	Generalisability	That is, whether the results apply to wider patient groups (and over a longer follow-up), Asian populations, and to routine clinical practice in the local context	Generalisability/External Validity
EUnetHTA European Network for Health Technology Assessment, Europe [18]	HTA Core Model Version 3.0, 2016	generic (diagnostic technologies, medical interventions, surgical interventions, pharmaceuticals, screening technologies)	Transferability, generalisability	The extent to which the results of existing studies are likely to reflect the results expected in the population of interest in different jurisdictions or health systems	Transferability
GÖG Gesundheit Österreich GmbH and LBI Ludwig Boltzmann Institut, Austria [19]	Methodenhandbuch für Health Technology Assessment Version 1.2012, 2012	generic (health technologies)	Generalisability	Generalisability refers to the question, whether study results can be applied to another context [translated]	Generalisability/External Validity
HIQA Health Information and Quality Authority, Ireland [20]	Guidelines for the Retrieval and Interpretation of Economic Evaluations of Health Technologies in Ireland, 2014	generic (“drugs, equipment, diagnostic techniques and health promotion activities”)	Generalisability, transferability, transportability, external validity, relevance or applicability	The extent to which one can apply or extrapolate results obtained in one setting or population to another	Generalisability/External Validity
HQA Health Quality Ontario, Canada [21]	Health Technology Assessments – Methods and Process Guide Version 2.0, 2018	non-pharmaceuticals (e.g. medical device and health care services (e.g. medical devices, medical tests, surgical procedures, health care programs, complex health system interventions)	Applicability	NR^b^	NA^a^
			Generalisability	Generalisability refers to ‘the problem of whether one can apply or extrapolate results obtained in one setting or population to another.’	Generalisability/External Validity
NICE National Institute for Health and Care Excellence, England and Wales [22]	Developing NICE guidelines: the manual (PMG20), 2014	generic (“from preventing and managing specific conditions, improving health, and managing medicines in different settings, to providing social care and support to adults and children, and planning broader services and interventions to improve the health of communities”)	Applicability	How well an observation or the results of a study or a review are likely to hold true in a particular setting/non-study settings/for another population or in a different context	Transferability
SBU Swedish Agency for Health Technology Assessment and Assessment of Social Services, Sweden [23]	Assessment of methods in healthcare – a handbook, 2018	generic (all interventions involving prevention, diagnosis, treatment or care)	Transferability	NR	NA

^a^*NA* Not assignable

^b^*NR* Not reported

### Review purpose

SR-HEs can have several purposes. Four organisations [17, 21–23] use them to determine the need to conduct a P-HE. They consider a SR-HE sufficient if one or more P-HEs without major limitations and sufficient transferability are identified. If deemed necessary, the P-HEs are updated and/or adapted to the decision context. In contrast, GÖG/LBI questions whether a SR-HE can be used to answer questions on cost-effectiveness, because transferability is often limited. That said, GÖG/LBI [19] acknowledges that SR-HEs are useful to explore how and why interventions may be more or less effective, resource or cost intensive and to collect important information for performing a P-HE. EUnetHTA [18] suggests using SR-HEs if a P-HE cannot be conducted (eg, due to limited resources). In this case SR-HEs can be used for explanatory purposes or to identify the most relevant P-HE for informing a particular decision.

### Assessment approach concepts

We developed definitions for different aspects of the assessment approach concepts to describe how the transferability assessment is recommended to be operationalised and provide these in Table 1. An overview of the recommended assessment approach concepts can be found in Table 3. Further details are available as supplement (see Additional file 2).

**Table 3:** Assessment approach concepts

Organisations	Target data (Consideration of transferability for:)		Integration of assessment in the preparation process (Consideration of transferability in:)		Structuring of assessment approach		Guidance on completion provided		Combination of different assessments		Consideration of results from sensitivity analyses	
	Effectiveness data	Cost data NR	In study selection	In study assessment	non-structured	structured	yes	no	Stand-alone	combined	yes	NR
ACE [17]	NR^a^	y	x	x		x		x	x		x	
EUnetHTA [18]	NR	x		x	x			x	x			x
GÖG/LBI [19]	x	x	x	x		x	x		x		x	
HIQA [20]	x	x	x	x		x	x		x		x	
HQA [21] (Applicability)	NR	x	x	x		x		x	x			x
HQA [21] (Generalis-ability)	NR	x		x	x			x	x			x
NICE [22]	x	x	x	x		x	x		x		x	
SBU [23]		x		x		x		x		x		x

^*a*^*NR* Not reported

Aspects of transferability can be considered at different steps during the preparation of a SR-HE: At the selection-level, when defining and applying eligibility criteria and at the assessment-level, when assessing the studies quality (and transferability). All included organisations [17–23] recommend a transferability assessment at the assessment level. Five organisations [17, 19–22] additionally recommend to consider aspects of transferability at the selection level. Moreover, NICE [22] proposes to limit the study setting to the UK or countries with similar healthcare systems, if necessary.

The assessment approaches vary regarding the target data for assessing transferability, the assessment structure, the provision of guidance for completion, the combination of different assessments (transferability and methodological quality) and the consideration of results derived from sensitivity analyses.

#### Target data

Regarding the target data for assessing transferability, three organisations [19, 20, 22] consider the transferability of cost and effectiveness data. One organisation [23] considers the transferability of cost data. For three organisations [17, 18, 21] it is unclear, whether their recommendations consider only cost data, or both.

#### Structuring of the assessment approach

Regarding the assessment structure, six [17, 19–23] concepts can be defined as structured approaches, comprising for example checklists or questionnaires. Two concepts are non-structured approaches, which may include examples for potentially relevant assessment criteria [18, 21].

#### Guidance on completion

Regarding the provision of guidance in the form of instructions or item descriptions for assessors, four organisations [17, 18, 21, 23] do not provide any instructions or item descriptions, while three organisations [19, 20, 22] provide some. These mainly include explanations and examples of assessment criteria, explanations and examples why something might have an impact on transferability and/or guidance on what should be considered in the assessment.

#### Combination of different assessments

Regarding the combination of different assessments, six organisations [17–22] recommend assessing transferability independently from other aspects (standalone assessment), e.g. the methodological quality while one organisation [23] recommends a combined assessment. Specifically, SBU [23] recommends combining the assessment of transferability and methodological quality, including a joint overall judgement. Although NICE [22], HQA [21] and HIQA [20] recommend a standalone assessment, they link the assessments of transferability and methodological quality: NICE [22] and HQA [21] recommend to assess the methodological quality only if the assessed study is sufficiently transferable, while HIQA [20] recommends to assess transferability only for studies with acceptable quality.

#### Consideration of results from sensitivity analyses

Four [17, 19, 20, 22] organisations recommend to consider information derived from sensitivity analyses in the context of transferability assessments. For example, according to ACE, “any key drivers of the economic model and areas of uncertainty identified by the sensitivity analysis should be included in the evaluation report.” [17].

### Assessment criteria

The assessment criteria describe which factors might affect transferability and are recommended by HTA organisations for consideration in transferability assessments. An overview of all recommended assessment criteria is provided in Table 4. More details can be found as supplement (see Additional file 3).

**Table 4:** Assessment criteria

Domain	Items	ACE [17]	EUnetHTA [18]	GÖG/LBI [19]	HIQA [20]	HQA [21] (Applic-ability)	HQA [21] (Generalis-ability)	NICE [22]	SBU [23]
Population	Population (in general)	x				x		x	
	Demographics		x	x	x		x		
	Risk factors		x						
	Life expectancy		x	x					
	Compliance		x	x					
	Ethnicity				x				
	Epidemiology		x	x	x		x		
	Stage/severity of disease		x						
	Case mix		x	x					
	Variation in health state values		x	x	x		x		
Intervention	Intervention (in general)	x				x		x	
	Extent and type of care								x
Comparator	Comparator (in general)	x							
Outcome	Health state preferences (e.g. in terms of QALYs)				x	x		x	
	Appropriateness of measures					x		x	
	Source of estimates of treatment effects					x			
Health system	Health system (in general)	x	x			x		x	
	Availability of health technologies			x					
	Available treatment options		x						
	Incentives for healthcare professionals or institutions				x		x		
	Resource availability	x			x		x		
Clinical practice	Clinical practice (in general)	x		x	x		x		
	Clinical guidelines				x				
	Care pathways				x				
	Treatment practice				x				
	Range of treatment				x				
	Provider characteristics		x						
	Provision of health services by different professional groups			x					x
	Organisation of prescribing			x					
Costs	Completeness of cost data	x							
	Unit prices/costs		x						x
	Absolut and relative prices/costs			x					
	Medical costing approach			x					
	Relative prices/costs				x		x		
	Value of various cost elements				x				
Methodo-logical aspects	Perspective	x	x	x	x	x		x	x
	Discount rate		x	x	x	x		x	
	Modelling approach				x				
	Assuming effects to be equal								x
	Extrapolation				x				
Other	Clinical effectiveness data				x				

#### PICO (Population, Intervention, Comparison, Outcome)

With exception of SBU [23], all organisations consider population characteristics in their recommended assessments [17–22]. Four organisations [18–21] take demographic characteristics, epidemiology (e.g., incidence/prevalence) and values of health sate preferences into account. EUnetHTA [18] and GÖG/LBI [19] additionally consider life expectancy, compliance and comorbidities. Other items are risk factors [18], severity of disease [18], ethnicity [20] and case mix [19].

Four organisations consider intervention characteristics. Three of them [17, 21, 22] pose the question, of whether the intervention is appropriate for the research question of the SR-HE. SBU [23] recommends to compare the care and type of intervention with the current intervention in the target context. ACE [17] additionally includes a question regarding the similarity between the study’s comparator and the comparator proposed in the research question.

Three organisations consider outcome characteristics [20–22]. All of them take health state preferences in terms of QALYs into consideration. Furthermore, HQA [21] and NICE [22] consider the appropriateness of measures. HQA [21] also checks whether estimates of treatment effects are from the best available source.

#### Health system

Health system characteristics are considered by six [17–22] organisations. Three of them pose the question of whether the health system, in which the study was conducted, can be compared to that of the context of interest [17, 21, 22]. Others recommend to examine health system characteristics like available treatment options and unit prices [18], availability of the health technology of interest [19] or incentives to healthcare professionals and institutions [20, 21].

#### Clinical practice

Six organisations [17–21, 23] consider variation in clinical practice. Some of them specify aspects including provider characteristics [18], treatment practice [20], clinical guidelines [20], care pathways [20], range of treatments [20], organisation of prescribing [19] and provision of health services by different professional groups [19, 23].

### Costs

Six [17–21, 23] out of seven organisations recommend the consideration of cost data. The following items are suggested: completeness of cost data [17], unit prices/costs [18, 23], absolute and relative prices/costs [19], the medical costing approach [19], relative prices/costs [20, 21] or the value of various costing elements (e.g. charges or fees) [20].

#### Methodological aspects

All organisations consider methodological aspects of the studies [17–23], in particular a study’s perspective which determines the costs and consequences that were considered in the analysis [17–23]. Five organisations [18–22] also consider the appropriateness of discounting. Moreover, HIQA [20] considers the appropriateness of the model used to extrapolate data to the context of interest, while SBU [23] questions whether costs and effects were studied or whether effects were assumed to be equal.

## Discussion

Our review summarises the methodological recommendations of seven HTA organisations for considering transferability in the context of SR-HEs. However, the few hits of our structured search show that this topic still receives little attention in methodological recommendations. In accordance, a review of Mathes et al. [13] identified 13 HTA methods documents addressing SR-HEs, of which only four included recommendations for assessing transferability. According to Luhnen et al. [24], who analysed the methods applied for SR-HE in published HTAs, only 10% of SR-HEs included in HTA reports include an assessment of transferability.

### Review purpose

The included organisations have different objectives when performing a SR-HE. For example, GÖG/LBI [19] use them in an exploratory way, while other organisations recourse to identified PH-Es for cost-effectiveness evaluations as far as they do not have any major limitations and sufficient transferability. This may be the case because the different organisations take different stances on the transferability of P-HE: some accept them and consider their limitations, other deem their use generally inappropriate. Not surprising, these general judgements seem to impact the overall approach and related recommendations for considering transferability. That said, the assessment approach concepts and assessment criteria also differ between organisations with similar views on the usefulness of SR-HEs. Some differences might be explained by the varying degree of details/item descriptions provided.

### Assessment approach concepts

We identified various concepts for assessing transferability in the context of SR-HEs. It is conspicuous that none of the included HTA organisations recommends a previously published tool without adoptions (only HIQA [20] recommends the use of “any” published tool), suggesting that there is no widely accepted tool. There are several potential reasons for this. For example, some published tools vary in scope and content (e.g. combing methodological study quality, reporting quality, transferability) compared to recommended approaches of HTA organisations [3]. Further, complexity and expenditure of time might limit feasibility of tools for practical application in preparing HTAs. Kim et al. [25] also question the suitability of these tools for local authorities due to their technical and complex nature. Therefore, they developed a decision framework and practical guidance, which might better suit the specific requirements for preparing evidence for local decision making.

The recommendations differ regarding various aspects of their assessment approach concepts (e.g. assessment structuring or target data). All organisations recommend the consideration of transferability in the study assessment. More than half of the organisations also recommend considering aspects of transferability as an eligibility criterion [17, 19–22]. However, in most cases there is no clear guidance when (e.g. whether studies are directly excluded in the selection process, or post-hoc after performing the study assessment) and which (i.e. what means insufficient) studies should be excluded due to transferability reasons.

Most recommended assessment approach concepts are structured (e.g. recommending a checklist, questionnaire or successive steps for the assessment). Comparing these assessment approach concepts with published tools for assessing transferability (identified through systematic reviews from Goeree et al. [3] and Munthe-Kaas et al. [26]) reveals some differences. For example, Welte et al. [11] and Drummond et al. [6] developed decision charts for assessing the transferability of economic evaluation results, but none of organisations recommends a decision chart. Further, Boulenger et al. [10] developed a checklist, including a score which represents the percentage of checklist items that were adequately or partially addressed in the study, although such a score was not recommended by any HTA organisation.

Regarding the combination of assessment approaches (transferability and methodological study quality), all except one organisations recommend to assess transferability separately from methodological study quality. Nevertheless, three organisations [20–22] link the assessments of transferability and methodological quality by making them interdependent, i.e. by only assessing methodological quality for studies with acceptable transferability or vice versa. Comparable recommendations can be found for published tools: According to Späth et al. [27] assessors should judge whether methodological minimum requirements are met before assessing transferability. Welte et al. [11] and Drummond et al. [6] consider insufficient methodological quality as a knock-out criterion. Moreover, Antonanzas et al. [9] recommend to evaluate the methodological study quality initially as a part of the “general transferability index”. Here, a poor rating for several methodological quality aspects can lead to a rating as generally non-transferable. Thus, all these tools put the assessment of methodological study quality first. Interestingly, only one HTA organisation recommends to assess transferability solely for studies with acceptable quality [20], while two organisations recommend the opposite [21, 22], that is to assess methodological study quality solely for studies with sufficient transferability. Furthermore, these approaches are contrary to recommendations for preparing effectiveness reviews. In case of effectiveness reviews there is no acknowledged guidance that recommends the exclusion of studies due to limited study quality [28–30].

### Assessment criteria

The recommendations include various assessment criteria. Overall, the assessment criteria are heterogeneous and vary in scope and content. We found no hint that differences in terminology would explain this heterogeneity.

We assigned the different assessment criteria (in form of items) to the following domains: population, intervention, comparator, health system, clinical practice, costs and methodological aspects. Comparing these domains and the assigned items between the different organisations shows that the items vary between the organisations, while the domains have a significant overlap. This might be due to the fact that several organisations provide broad assessment criteria, while others include more details. The application of general and unspecific assessment criteria might be explained by the broad scope of the transferability assessment, concrete that the guidance of most HTA organisations refer to all types of health technologies. In this case it is difficult to find a good balance between level of detail and applicability across different types of health technologies. However, unspecific assessment criteria in conjunction with missing instructions leave plenty of scope for user interpretation, which might in turn lead to unsystematic or inconsistent assessments. Only three out of seven organisations provide guidance on completion to support assessors [19, 20, 22].

We further compared the assessment criteria included in the recommended assessment approaches with those of tools from Drummond et al. [6], Welte et al. [11] and Antonanzas et al. [9] and with empirically assessed criteria, identified by the review of Sculpher et al. [7]. The comparison shows heterogeneity both between published tools and regarding the assessment criteria recommended by different HTA organisations. In particular, it is conspicuous that all published tools include the intervention and comparator treatment as assessment criteria, while these are less frequently recommended by the HTA organisations. A possible explanation would be that these aspects are already considered by the HTA organisations when defining and applying eligibility criteria and therefore are not considered in the study assessment. Nevertheless, we identified some assessment criteria that were recommended by the majority of included HTA organisations and in addition by the previously published tools and the empirical evidence. These include demographics, epidemiology, health state preferences, healthcare system (in general), clinical practice (in general), perspective and discount rate.

### Limitations

This review is not without limitations. First, we only considered English and German language methods documents. Second, we did not contact HTA organisations for unpublished documents Thus, we were also not able to clarify unspecific and insufficient descriptions. Third, our literature search was conducted in 2019. However, methodological developments take time and HTAs methods documents are usually updated infrequently. Therefore, it can be assumed that there have been no major changes in this context since then.

## Conclusion

Different approaches exist on how to consider transferability of P-HEs, when performing a SR-HE. Some tools have already been published and suggested [3]. However, the included HTA organisations mainly recommend using their own or adapted tools for assessing transferability. There is no commonly used approach/tool for transferability assessments.

The methodological recommendations differ regarding assessment approach concepts and assessment criteria. The structure (e.g, checklist, questionnaire), the step in the SR-HE preparation process, at which transferability should be considered and the link between transferability and methodological study quality also vary between HTA organisations, as well as compared to previously published tools. Differences can also be observed regarding the assessment criteria.

Transferability considerations may depend on the review purpose and should fit the relevant medical area and specific decision contexts. Obviously, generic guidance for all types of health technologies must always allow flexibility to be applicable to the different types of health technologies. A solution might be the development of an assessment tool which comprises a set of core items (assessment criteria which are relevant to most research questions and health technologies) and additional ‘add on’ items (assessment criteria which are only relevant to specific research questions or health technologies, e.g. diagnostics or public health interventions). Regardless of whether the items are core or add-on items, a context-specific formulation, instead of standardised questions would be necessary. Moreover, because of this complexity and inherent heterogeneity, the provision of instructions, explanations and examples appears to be of particular importance. The generated list of assessment criteria provides a comprehensive overview of potentially relevant criteria for assessing transferability. The list might be used as a starting point for determining the relevant items of a transferability assessment tool (e.g, core and add-on items) or when determining the relevant criteria that might affect transferability in a particular decision problem. There are some assessment criteria, which were considered by the majority of included HTA organisations and by the previously published tools and empirical evidence [6, 7, 9, 11]. This suggests that these might have the potential be core items in future assessment tools.

## Supplementary Information

**Additional file 1.** List of Excluded Documents.

**Additional file 2.** Assessment Approach Concepts.

**Additional file 3.** Assessment Criteria.

## Data Availability

All data generated or analysed during this study are included in this published article and its supplementary information files.

## Abbreviations

ACE: Agency for Care Effectiveness
EUnetHTA: European Network of HTA Agencies
GÖG: Gesundheit Österreich GmbH
HIQA: Health Information and Quality Authority
HQA: Health Quality Ontario
HTA: Health Technology Assessment
HTAi: Health Technology Assessment international
INAHTA: International Agency of Health Technology Assessment
LBI: Ludwig Boltzmann Institut
NICE: National Institute for Health and Care Excellence
P-HE: Primary Health Economic Evaluation
PICO: Population, Intervention, Comparison, Outcome
PRISMA: Preferrred Reporting Items for Systematic Reviews and Meta-Analysis
QALYs: Quality Adjusted Life Years
RedETSA: Red de Evaluación de Tecnologías en Salud de las Américas
SBU: Swedish Agency for Health Technology Assessment and Assessment of Social Services
SR-HE: Systematic Review of Health Economic Evaluations
