# Supplementary material for: Assessing transferability in systematic reviews of health economic evaluations – a review of methodological guidance
Source: BMC Med Res Methodol. 2022 Feb 20;22:52. doi: 10.1186/s12874-022-01536-6 (PMC8858549; doi:10.1186/s12874-022-01536-6)
Supplement: Supplementary file 1 — Additional file 1. List of Excluded Documents. [file 12874_2022_1536_MOESM1_ESM.docx]

Table 1 List of excluded documents

| No. | Organisation (abbreviation) | Document title (year) | Main reason for exclusion |
| --- | --- | --- | --- |
|  | AZZ | The Croatian Guideline for Health Technology Assessment Process and Reporting (2011) | Transferability recommendations |
|  | ACE | Costing template for manufacturers | Publication type |
|  | ACE | Methods and process guide for drug evaluations (2018) | Transferability recommendations |
|  | AETS | Guia para la elaboracion de informes de evaluacion de tecnologias sanitarias | Language |
|  | AETS | Organizaction y funcionamiento de los comites autonomicos de evaluacion (…) | Language |
|  | AETSA | Criterios de calidad y buenas practicas en organismos dedicados a la evaluacion de tecnologias sanitarias | Language |
|  | AETSA | Elaboración de Guías de Práctica Clínica en el Sistema Nacional de Salud. Actualización del Manual Metodológico (Julio 2016) | Language |
|  | AETSA | Guía para la elaboracíon de informes de evaluación de medicamentos | Language |
|  | AETSA | Guía para la elaboracíon de informes de síntesis de evidencia: medicamentos | Language |
|  | AETSA | Guideline for the Elaboration and Adaptation of Rapid Health Technology Assessment Reports (April 2016) | Language |
|  | AETSA | Guideline for the elaboration of recommendations and appropriate use criteria in health technologies | Language |
|  | AETSA | Red de Evalucacíon de tecnologías sanitarias en el SSPA. Metodologia (…) | Language |
|  | Agenas | Rapid Health Technology Assessment for Agneas | Transferability recommendations |
|  | Agenas | Manuale procedure HTA (2014) | Language |
|  | Agenas | Rapid Health Technology Assessment for Agneas | Language |
|  | Agneas | HTA Report Adaption: Documento metodologico (2013) | Language |
|  | AHRQ | Methods Guide for Effectiveness and Comparative Effectiveness Reviews (2014) | Transferability recommendations |
|  | AHRQ | Methods Guide for Medical Test Reviews (2012) | Transferability recommendations |
|  | AHS | Influencing Decision and Policy Making with HTA (2006) | Publication type |
|  | AHS | Overview of common issues and research methods used in HTA (2006) | Transferability recommendations |
|  | AHS | Putting HTA into Practice (2012) | Publication type |
|  | AHS | Background to health technology assessment (2012) | Duplicate |
|  | AHS | Where do you find information for health technology assessment? (2005) | Publication type |
|  | AHTAPol | Health Technology Assessment Guidelines Version 3.0 (2016) | Transferability recommendations |
|  | ASSR | Metodologia di ricerca bibliografica, selezione e valutazione delle evidenze per la redazione degli short report (2010) | Language |
|  | BAG | Faktenblatt Bewertung von Gesundheitstechnologien (HTA) (2016) | Publication type |
|  | BAG | Frascati Manual (2015) | Publication type |
|  | BAG | Priorisierungs-und ausschlusskriterien für HTA Themen (2018) | Publication type |
|  | CADTH | Guidelines for Authors of CADTH Health Technology Assessment (2003) | Transferability recommendations |
|  | CADTH | Guidelines for the Economic Evaluation of Health Technologies Canada 4th Edition (2017) | Transferability recommendations |
|  | CADTH | Indirect Evidence: Indirect Treatment comparisons in Meta-Analysis (2009) | Transferability recommendations |
|  | CADTH | Quality Assessment Tools Project Report (2012) | Publication type |
|  | CCATES | Methodological Guidelines Health Technology Performance Assessment (2017) | Publication type |
|  | CMTP | CED-Issue-Brief (2009) | Publication type |
|  | CMTP | Effectiveness Guidance Documents | Publication type |
|  | CONITEC | Diretrizes Metodológicas - Diretriz de Avalicao econômica (2014) | Language |
|  | CONITEC | Diretrizes Metodológicas - Elaboracao de Pareceres Técnic - Científico | Language |
|  | CONITEC | Diretrizes Metodológicas - Elaboracas de revisao sistematica e metanalise de estudos de acuracia diagnostica | Language |
|  | CRUF AOUIVR | Regolamento per lo svolgimento di studi profit e noprofit in aoui verona | Language |
|  | CUFAR | Manual de Procedimentos para los comités de farmacoterapéutica y seleccion de medicamentos (2009/2013) | Language |
|  | DDCTSD | Interrogantes daves a la hora de disenar un ente de evaluacion de tecnologias sanitarias: Respecestas desde tres perspectivas | Language |
|  | DDCTSD | Plan Nacional de ciencia y tecnologia en salud (2002-2010) | Language |
|  | DHA | Health Technology Assessment Handbook (2007) | Transferability recommendations |
|  | DHA | Introduction to Mini-HTA (2005) | Transferability recommendations |
|  | EL Salvadorian Ministry of Health | Manual de Organización y funciones de la Dirección de tecnologías sanitarias (2015) | Language |
|  | EUnetHTA | Handbook | Transferability recommendations |
|  | EUnetHTA | HTA adoption toolkit (2011) | Publication type |
|  | EUnetHTA | LEVELS OF EVIDENCE Applicability of evidence for the context of a relative effectiveness assessment (2015) | Transferability recommendations |
|  | Fimea (FINOSE) | Process for Joint Assessment (2018) | Publication type |
|  | Fimea | Rapid Assessment of New Hospital-Only Medicinal Products (2018) | Publication type |
|  | FinCCHTA | HTA opas versio 1.1 (2017) | Language |
|  | GBA | Verfahrensordnung des Gemeinsamen Bundesausschusses (letzte Änderung 01.11.2018) | Publication type |
|  | HAS | Choices in Methods for Economic Evaluation (2012) | Publication type |
|  | HAS | General Method for Assessing Health Technologies (2007) | Transferability recommendations |
|  | HAS | Rapid Assessment Method For Assessing Medical And Surgical Procedures (2007) | Transferability recommendations |
|  | HealthPACT | Researcher Uuser Guide (RUG) (2010) | Publication type |
|  | HIQA | A Guide to Health Technology Assessment at HIQA (2016) | Publication type |
|  | HIQA | Guidance on Budget Impact Analysis of Health Technologies in Ireland (2015) | Outdated |
|  | HIQA | Guideline for the Economic Evaluation of Health Technologies in Ireland (2018) | Transferability recommendations |
|  | HIQA | Guidelines for Evaluating the Clinical Effectiveness of Health Technologies in Ireland (2014) | Outdated |
|  | HIQA | Guidelines for the Budget Impact Analysis of Health Technologies in Ireland (2018) | Transferability recommendations |
|  | HIQA | Guidelines for Evaluating the Clinical Effectiveness of Health Technologies in Ireland (2018) | Transferability recommendations |
|  | HIS | Meetings in Public | Publication type |
|  | HIS | Draft Standard operating procedure for innovative medical technology overviews (2014) | Publication type |
|  | HIS | Framework for producing SHTG Advice Statements (2013) | Publication type |
|  | HIS | Standard operating procedure for production of horizon scanning reports (2014) | Publication type |
|  | HIS | Standard operating procedure for the production of evidence notes (2012) | Publication type |
|  | HIS | Standard operating procedure for the production of technologies scoping reports (2012) | Publication type |
|  | HVB | EBM und HTA Berichte in der Abteilung EWG (2018) | Transferability recommendations |
|  | IACS | Actualizaci´n de Guías de Práctica Clínica en el Sistema Nacional de Salud. Manual Metodológico (2009) | Language |
|  | IACS | IElaboración de Guías de Práctica Clínica en el Sistema Nacional de Salud. Actualización del Manual Metodologic (2016) | Language |
|  | IACS | Implicaión de Pacientes en el Desarrollo de Guías de Práctica Clínica. Manual Metodológico (2013) | Language |
|  | ICER | Metholología | Language |
|  | ICER | A Guide to ICER's Methods for Health Technology Assessment (2018) | Transferability recommendations |
|  | ICER | Icers reference Case for Economic Evaluations: Principles and Rationale (2018) | Publication type |
|  | ICER | ICER-Guide-to-Understanding-Health-Technology-Assessment-(2018) | Publication type |
|  | IETS | Guidelines for the economic evaluation of healthcare technologies in Columbia: Technical support documents (2014) | Publication type |
|  | IETS | Documentos técnicos de apoyo a la construccion de referencia colombiano para la evaluacion economica en salud (2014) | Language |
|  | IETS | Manual Metodologico - Evaluacion de tecnologias en salud (2014) | Language |
|  | IETSI-ESSALUD | Directiva para el Desarrollo de Guías de Práctica Clínica en Essalud (2016) | Language |
|  | IETSI-ESSALUD | Documento Tecnico Metodologá para la Elaboracón de Guias de Practica Clinica (2015) | Language |
|  |  | Implementatción de Guías de Práctica Clínica en el Sistema Nactional de Salud. Manual Metodológico (2009) | Language |
|  | IHE | Health Technology Assessment Program Process and Impact Evaluation Final Report (2011) | Publication type |
|  | INESS | Les normes de production des revues systematiques (2013) | Language |
|  | INAHTA | INAHTA Checklist for health technology assessment reports (2007) | Publication type |
|  | INEAS | Formulaire de Demande d'Évaluation des Technologies de Santé | Publication type |
|  | INEAS | Guide D’Acquisition d’une technologie innovante | Publication type |
|  | INESSS | Cadre processus et méthodes pour la réalisation du guide méthodologique d'elaboration et d'adaption des guides de pratique (2017) | Publication type |
|  | INESSS | Elaboration adaptation guides de pratique (2017) | Publication type |
|  | INFARMED | Guidelines for Economic Drug Evaluation Studies (1998) | Publication type |
|  | Institute of Population Health University of Ottawa | Taking account of context in population health intervention research (2018) | Transferability recommendations |
|  | IQWiG | Methoden 5.0 (2017) | Transferability recommendations |
|  | KCE | Process Book (2018) | Publication type |
|  | LBI | HTA (Externes) Manual Selbstverständnis und Arbeitsweise (2007) | Transferability recommendations |
|  | LBI | (Internes) Manual Abläufe und Methoden (2007) | Transferability recommendations |
|  | LBI | HTA Methodenhandbuch (2012) | Duplicate |
|  | MAHTAs | Health Technology Assessment (HTA) Manual | Transferability recommendations |
|  | MAHTAs | Manual on Development and Implementation of Evidence-Based Clinical Practice Guidelines | Transferability recommendations |
|  | MAHTAs | Manual on Horizon Scanning of Health Technologies | Publication type |
|  |  | Meeting in public Scottish Health Technology Group (2016) | Duplicate |
|  | Ministeri de Salud Costa Rica | Modelo Conceptual y Estragecio de Evaluation del Impacto (2012) | Language |
|  | Ministerio de Salud Costa Rica | Modelo Conceptual y Estrategico (2011) | Language |
|  | Ministerio de Salud Costa Rica | Modelo Conecptual Y Estrategico Del Instituto de Nutricion y Desarrollo Infantil (2017) | Language |
|  | MSAC | Assessment Report Template - Investigate | Publication type |
|  | MSAC | Assessment Report Template - Therapeutic | Publication type |
|  | MSAC | Medical Services advisory committee: Clinical utility card for heritable mutations which increase risk in (disease area) | Publication type |
|  | MSAC | Technical Guidelines for preparing assessment reports for the Medical Services Advisory Committee - Service Type: Investigate (2017) | Publication type |
|  | MSAC | Technical Guidelines for preparing assessment reports for the Medical Services Advisory Committee - Service Type: Therapeutic (2016) | Publication type |
|  | NCPE | Guidelines for Inclusion of Drug Costs in Pharmacoeconomic Evaluations (2018) | Publication type |
|  | NICE | Diagnostics Assessment Programme Manual (2011) | Transferability recommendations |
|  | NICE | Diagnostics Interim addendum Guidance Review | Publication type |
|  | NICE | Evidence summaries process guide (2017) | Publication type |
|  | NICE | Evidence Standards Framework for Digital Health Technologies (2019) | Publication type |
|  | NICE | Guide to the methods of technology appraisal | Publication type |
|  | NICE | Guide to the processes of technology appraisal | Publication type |
|  | NICE | Guide to the technology appraisal and highly specialised technologies appeal process (2014) | Publication type |
|  | NICE | Interim methods guide for developing good practice guidance (2014) | Publication type |
|  | NICE | Interim Process and Methods of the Highly Specialized Technologies (2011) | Outdated |
|  | NICE | Interventional prodecures Manual (2016) | Transferability recommendations |
|  | NICE | Medical technologies evaluation programme methods guide (2017) | Transferability recommendations |
|  | NICE | Methods for the development of NICE public health guidance (third edition) (2012) | Outdated |
|  | NICE | Methodology for developing costing tools | Publication type |
|  | NICE | Process guide for adoption support resources for health technologies (2015) | Publication type |
|  | NOMA | Guidelines for the submission of documentation for single technology assessment (STA) of pharmaceuticals (2018) | Publication type |
|  | NSPHMPDB | Romania Health system review (2016) | Publication type |
|  | OSTEBA | Evaluacion de tecnologias sanitorias (1999) | Language |
|  | OSTEBA | Proyetco metodologico - Sintesis de informacion relevante de apoyo a los MCDA para la toma de decisones (2019) | Language |
|  | Panama Ministry of Health | PROCESO DE AUDITORÍA SOCIAL DE LA CARTERA DE PRESTACIONES PRIORIZADAS (2014-2015) | Language |
|  | PBAC | Procedure guidance for listing medicines on the pharmaceutical benefits scheme (2019) | Publication type |
|  | PBAC&MSAC | Technical Guidelines for preparing assessment reports for the Medical Services Advisory Committee (Investigate) (2017) | Duplicate |
|  | PBAC&MSAC | Technical Guidelines for preparing assessment reports for the Medical Services Advisory Committee (Therapeutic) (2016) | Duplicate |
|  | PCORI | Methodology Report (2019) | Transferability recommendations |
|  | PCORI | Methodology Standards (2018) | Publication type |
|  | RCHD | СОВЕРШЕНСТВОВАНИЕ СИСТЕМЫ ОЦЕНКИ МЕДИЦИНСКИХ ТЕХНОЛОГИЙ (2017) | Language |
|  | SBU | Evaluation and synthesis of studies using qualitative methods of analysis (2016) | Publication type |
|  | SESCS | Metodologá para incorporar los estudio cualitativos en la evaluación de tecnologías sanitarias | Language |
|  | SESCS | Proceso de Incorporacion de tecnologias sanitarias (PITS) en el servisio canario de la salud | Language |
|  | SESCS | Propuesta de guía para la evaluación evonónmica aplicada a las tecnologías sanitarias | Language |
|  | SUKL | Cost-effectiveness analysis - critical appraisal procedure | Publication type |
|  | SUKL | Course of administrative procedure for determination of the maximum price (…) (2018) | Publication type |
|  | SUKL | Procedure for processing an application (…) (2018) | Publication type |
|  | TLV | Economic evaluation of medical devices final report 2015 | Publication type |
|  | VASPVT | Background Analysis for National HTA Strategy for Lithuania - Focus on Medical Devices (2015) | Publication type |
|  | ZIN | Criteria voor beoording therapeutische waarde | Language |
|  | ZIN | Guideline for economic evaluations in health care (2016) | Publication type |
|  | ZIN | Kostenhandleiding Methodologie van Kostenanderzoek (…) | Language |
|  | ZIN | Procedure beoordeling extramurale geneesmiddelen (2018) | Language |
|  | ZIN | Richtlijn voor het uitvoreren van economische evaluatries in de gezondheidszorg | Language |
|  | ZonMw | Summary assessment procedure (2012) | Publication type |
