# Supplementary material for: Assessing transferability in systematic reviews of health economic evaluations – a review of methodological guidance
Source: BMC Med Res Methodol. 2022 Feb 20;22:52. doi: 10.1186/s12874-022-01536-6 (PMC8858549; doi:10.1186/s12874-022-01536-6)
Supplement: Supplementary file 2 — Additional file 2. Assessment Approach Concepts. [file 12874_2022_1536_MOESM2_ESM.docx]

| **Organisation** | **Description of Assessment Approaches** |
| --- | --- |
| ACE | - Own questionnaire for assessing applicability of economic evaluations: |
|  | - Consideration of assessment criteria according to population, intervention, comparator, health system, clinical practice, costs, methodological aspects |
| GÖG/LBI | - Systematic assessment of the transferability of economic evaluations: |
|  | - Analyzing the relevance of 'final transferability factors' (according to Welte et al, 2004) for each study |
|  | - Assessing the consistency between the study country and the decision country (for each transferability factor) |
|  | - Description of consistency, under- or overestimation of the cost-effectiveness-relation between the study country and the decision country for transferability data |
|  | - If necessary: Adaption of study results, qualitative description of transferability aspects, application of transferability eligibility criteria or conduction of a country specific economic evaluation |
|  | - Consideration of assessment criteria according to population, health system, clinical practice, costs, methodological aspects and other |
| EUnetHTA | - List of factors potentially affecting transferability of economic data: |
|  | - Consideration of assessment criteria according to population, health system, clinical practice, costs, methodological aspects |
| HIQA | - Assessment of transferability of health economic evaluations: |
|  | - The transferability of the study results should be considered for all relevant studies that have an acceptable quality |
|  | - This should be conducted using a defined framework (e.g. EUnetHTA Core Model, Welte model, ISPOR model) |
|  | - Any reasons for lack of transferability should be clearly documented and any expected differences in the Irish setting explicitly stated |
|  | - Consideration of (key) assessment criteria according to population, outcome, health system, clinical practice, costs, methodological aspects and other |
| HQA | - Assessment of applicability of economic evaluations: |
|  | - Application of a modified applicability checklist for economic evaluations originally developed by NICE |
|  | - Possible responses of the checklist-questions: yes, partially, no, unclear, not applicable |
|  | - Possible overall judgements are: directly applicable, partially applicable, not applicable |
|  | - Consideration of assessment criteria according to population, intervention, outcome, health system, methodological aspects |
|  | - List of factors to consider when determining the generalisability of economic evaluations to the Ministry of Health and Long-Term Care context: |
|  | - Consideration of assessment criteria according to population, health system, clinical practice, costs |
| NICE | - Assessment of the applicability of economic evaluations as a part of the critical appraisal questionnaire: |
|  | - Section 1 of the 'appraisal checklist: economic evaluations' |
|  | - Possible answers: yes, partly, no, unclear, or NA (not applicable) |
|  | - Possible overall judgements are: directly applicable, partially applicable, not applicable |
|  | - Consideration of assessment criteria according to population, intervention, outcome, health system and methodological aspects |
| SBU | - Assessment of the transferability of the study's economic results as a part of the quality assessment: |
|  | - Two checklists (for assessing the quality of health economic modelling studies and for assessing the quality of trial based health economic studies) with equivalent transferability sections |
|  | - Possible transferability ratings are: high, moderate, low, insufficient |
|  | - Consideration of assessment criteria according to intervention, clinical practice, costs, methodological aspects |
| HQA | - List of factors to consider when determining the generalisability of economic evaluations to the Ministry of Health and Long-Term Care context: |
|  | - Consideration of assessment criteria according to population, health system, clinical practice, costs |
