# Supplementary material for: Assessing transferability in systematic reviews of health economic evaluations – a review of methodological guidance
Source: BMC Med Res Methodol. 2022 Feb 20;22:52. doi: 10.1186/s12874-022-01536-6 (PMC8858549; doi:10.1186/s12874-022-01536-6)
Supplement: Supplementary file 3 — Additional file 3. Assessment Criteria. [file 12874_2022_1536_MOESM3_ESM.docx]

| **Domain** | **Organisation** | **Item** |
| --- | --- | --- |
| Population | ACE | - Is the study population similar to this proposed in the research question? |
|  | EUnetHTA | - Factors potentially affecting transferability of economic data: |
|  |  | - Patient characteristics (demographics, risk factors, life expectancy, compliance, utilities) |
|  |  | - Disease characteristics (e.g. epidemiology, disease severity, case mix) |
|  |  | - Population characteristics (e.g. variations in the health state values used to form quality weights for the calculation of QALYs) |
|  | GÖG/LBI | - Factors affecting the generalisability of health economic evaluations: |
|  |  | - Life expectancy |
|  |  | - Case mix (disease risk, complication risk, remaining lifetime, demographical structure, gender structure, severity of illness, complication rate) |
|  |  | - Demographics |
|  |  | - Incidence and prevalence of the disease |
|  |  | - Spread of disease |
|  |  | - Effects of acceptance, compliance and incentive effects on the use of healthcare services |
|  | HIQA | - Factors to consider when assessing transferability: |
|  |  | - Population values (value of health state preferences may vary by demographic area |
|  |  | - Epidemiology (e.g. age structure of the population, incidence of various diseases) |
|  |  | - Specific concerns in relation to the transferability of clinical and economic data HTAs or clinical guidelines in the Irish healthcare setting: |
|  |  | - The generalisability of the economic and clinical data across different patient populations (e.g. age, gender, ethnicity) within Ireland |
|  | HQA | - Questions of the Study Applicability Appraisal Checklist: |
|  |  | - Is the study population similar to the question? |
|  |  | - Relevant Factors when determining generalisability: |
|  |  | - Population demography |
|  |  | - Disease epidemiology |
|  |  | - Health state preferences |
|  | NICE | Is the study population appropriate for the review question? |
| Intervention | ACE | - Is the intervention similar to this proposed in the research question? |
|  | HQA | - Questions of the Study Applicability Appraisal Checklist: |
|  |  | - Are the interventions similar to the questions? |
|  | NICE | - Are the interventions appropriate for the review question? (e.g. considering routine and best practice in UK settings) |
|  | SBU | - Do the extent and type of care or intervention delivered to the study participants correspond to what patients/users receive in the current Swedish context? |
| Comparator | ACE | - Is the comparator similar to this proposed in the research question? |
| Outcome | HIQA | - Factors to consider when assessing transferability: |
|  |  | - Differences in health state preferences in terms of QALYs |
|  | HQA | - Questions of the Study Applicability Appraisal Checklist: |
|  |  | - Are estimates of treatment effects from the best available source? |
|  |  | - Is the value of health effects expressed in terms of QALYs? |
|  |  | - Are outcomes from other sectors fully and appropriately measured and valued? |
|  | NICE | - Are QALYs derived using NICE's preferred methods, or an appropriate social care-related equivalent used as an outcome? |
| Health System | ACE | - Is the health system in which the study was conducted similar to the Singapore's context? |
|  |  | - Are estimates of treatment effects likely to be realized in the local context, taking into consideration resource availability? |
|  | EUnetHTA | - Factors potentially affecting transferability of economic data: |
|  |  | - Health system characteristics (e.g. available treatment options or unit prices) |
|  | GÖG/LBI | - Factors affecting the generalisability of health economic evaluations: |
|  |  | - Availability of the health technology (e.g. learning effects, geographical localization) |
|  | HIQA | - Factors to consider when assessing transferability: |
|  |  | - Incentives to healthcare professionals and institutions (e.g. physicians fee-for-service or salary, hospital input or output based) |
|  |  | - Availability of health resources |
|  | HQA | - Questions of the Study Applicability Appraisal Checklist: |
|  |  | - Is the health system in which the study was conducted similar to the current Ontario context? |
|  |  | - Relevant factors for determining generalisability: |
|  |  | - Incentives to health care professional and institutions |
|  |  | - Availability of health care resources |
|  | NICE | - Is the system in which the study was conducted sufficiently similar to the current UK context? |
| Clinical practice | ACE | - Are the estimates of treatment effects likely to be realized in the local context, taking into consideration variation in clinical practice? |
|  | EUnetHTA | - Factors potentially affecting transferability of economic data: |
|  |  | - Provider characteristics (e.g. clinical practice or quality of care) |
|  | GÖG/LBI | - Factors affecting the generalisability of health economic evaluations: |
|  |  | - Practice variation (e.g. organization of prescribing, provision of health services by different professional groups) |
|  | HIQA | - Factors to consider when assessing transferability: |
|  |  | - Variation in clinical practice (range of treatments, clinical guidelines, treatment setting, care pathway) |
|  |  | - Specific concerns in relation to the transferability of clinical and economic data to HTAs or clinical guidelines to the Irish healthcare setting: |
|  |  | - The generalisability of data due to local and regional differences in healthcare practice within Ireland |
|  | HQA | Relevant factors for determining generalisability: |
|  |  | - Variations in clinical practice |
|  | SBU | - Is the intervention implemented in a sector or by an organizsation (e.g. hospital care or local social service office) that is relevant to the current Swedish context? |
| Costs | ACE | - Are all relevant costs and consequences considered and included? |
|  | EUnetHTA | - Factors potentially affecting the transferability of economic data: |
|  |  | - unit prices |
|  | GÖG/LBI | - Factors affecting the generalisability of health economic evaluations: |
|  |  | - Absolut and relative prices |
|  |  | - Medical costing approach (e.g. different data sources) vs. productivity approach (e.g. human capital approach vs. friction costs) |
|  | HIQA | - Factors to consider when assessing transferability: |
|  |  | - Relative prices and costs |
|  |  | - Value of various cost elements (charges or fees, per diem costs, market prices, the approach to the inclusion of overheads, capital costs and maintenance) |
|  | HQA | - Relevant factors for determining generalizability: |
|  |  | - Relative costs |
|  | SBU | - Are the unit costs used in the study relevant to the current Swedish context? |
| Methodological aspects | ACE | - Is the perspective taken appropriate to the local context? |
|  | EUnetHTA | - Factors potentially affecting transferability of economic data: |
|  |  | - Study perspective (defines which costs and consequences are included in the analysis) |
|  |  | - Discount rate |
|  | GÖG/LBI | - Factors affecting the generalisability of health economic evaluations: |
|  |  | - Perspective (e.g. social insurance vs. society)  - Loss in productivity and working time |
|  |  | - Discount rate |
|  | HIQA | - Factors to consider when assessing transferability: |
|  |  | - Perspective (e.g. public payer, individual society) |
|  |  | - Appropriate discount rate |
|  |  | - Modelling approach: Consideration should be given whether the model structure, the assumptions which underpin it, and all data inputs adequately reflect the Irish situation |
|  |  | - Time horizon: Would the assumptions for extrapolation modelling differ in the context of interest? |
|  | HQA | - Questions of the Study Applicability Appraisal Checklist: |
|  |  | - Was/were perspective(s) clearly stated, and what were they? |
|  |  | - Are future costs and outcomes discounted? |
|  | NICE | - Is the perspective for costs appropriate for the review question? |
|  |  | - Is the perspective for outcomes appropriate for the review question? |
|  |  | - Are all future costs and outcomes discounted appropriately? |
|  | SBU | - Does the study have a societal perspective? |
|  |  | - Are both costs and effects studied (or are effects assumed to be equal)? |
| Other | HIQA | - Factors to consider when assessing transferability: |
|  |  | - Clinical effectiveness: |
|  |  | - Is the study population that the clinical effectiveness data was based on sufficiently similar? |
|  |  | - Are compliance rates reasonable? |
|  |  | - Is the extent to which the clinical efficacy data is representative of the likely effectiveness that can be achieved on Ireland |
